# Genetic Variation in Natural and Induced Antibody Responses in Layer Chickens

**DOI:** 10.3390/ani14111623

**Published:** 2024-05-30

**Authors:** Jesus Arango, Anna Wolc, Jeb Owen, Kendra Weston, Janet E. Fulton

**Affiliations:** 1 Hy-Line International, Dallas Center, IA 50063, USA; jesus.arango@cobbgenetics.com (J.A.); jfulton@hyline.com (J.E.F.); 2 Cobb Genetics, Siloam Springs, AR 72761, USA; 3 Department of Animal Science, Iowa State University, Ames, IA 50011, USA; 4 Department of Entomology, Washington State University, Pullman, WA 99164, USA; jowen@wsu.edu (J.O.); kendra.weston@wsu.edu (K.W.)

**Keywords:** natural antibody, induced antibody, GWAS, layer chicken

## Abstract

**Simple Summary:**

Improving the health of chickens is becoming of increasing importance as consumer expectations of cage-free and free-range housing result in more potential disease challenges while approved antibiotic treatments are decreasing. Antibodies are part of the immune response of chickens. Natural antibodies are present even though the bird has never been exposed to the specific antigen, and adaptive antibodies are produced after vaccination or exposure to a pathogen. This study looks for regions of the genome that influence both natural and adaptive antibody levels in three different breeds of commercially utilized egg production chickens. The genome regions that resulted in differential antibody-level production were identified with a high incidence of immune-related genes. Genetic influence (heritability) for antibody levels was also found. However, the genome regions and the level of heritability were different for different lines and different antigens, suggesting that there are not just a small number of genetic factors that influence overall antibody production. This study provides supporting evidence that overall health of chickens can be improved by genetic selection, though the exact genes and genomic regions still require further investigation, especially considering that the specific region can vary for different antigens and different chicken lines.

**Abstract:**

Selection of livestock for disease resistance is challenging due to the difficulty in obtaining reliable phenotypes. Antibodies are immunological molecules that provide direct and indirect defenses against infection and link the activities of both the innate and adaptive compartments of the immune system. As a result, antibodies have been used as a trait in selection for immune defense. The goal of this study was to identify genomic regions associated with natural and induced antibodies in chickens using low-pass sequencing. Enzyme-linked immunosorbent assays were used to quantify innate (natural) antibodies binding KLH, OVA, and PHA and induced (adaptive) antibodies binding IBD, IBV, NDV, and REO. We collected plasma from four White Leghorn (WL), two White Plymouth Rock (WPR), and two Rhode Island Red (RIR) lines. Samples numbers ranged between 198 and 785 per breed. GWAS was performed within breed on data pre-adjusted for Line-Hatch-Sex effects using GCTA. A threshold of *p* = 10^−6^ was used to select genes for downstream annotation and enrichment analysis with SNPEff and Panther. Significant enrichment was found for the defense/immunity protein, immunoglobulin receptor superfamily, and the antimicrobial response protein in RIR; and the immunoglobulin receptor superfamily, defense/immunity protein, and protein modifying enzyme in WL. However, none were present in WPR, but some of the selected SNP were annotated in immune pathways. This study provides new insights regarding the genetics of the antibody response in layer chickens.

## 1. Introduction

The poultry industry is in the middle of an important transformation in response to shifting public perceptions about animal welfare and the voluntary and regulatory initiatives to promote it. Two areas of interest are housing and production systems. Egg production is becoming more common in large and heavily populated cage-free environments around the world [1]. There is a growing consensus about the importance of producing more robust laying hens that can adapt to these evolving management styles, different housing systems, and environmental challenges, while maintaining good egg production and health [2,3,4]. With these industry changes, exposure to infectious disease is more common [5]. The tools available to prevent and control diseases in poultry are limited due to cost, large numbers of birds, inefficiency of anti-viral agents and risk of developing antimicrobial resistance [6], and they mainly rely on a fully functional and well-developed immune response. The immune system of poultry is complex; it includes both innate and adaptative responses that are composed of cellular and molecular effectors that coordinate to recognize, label, and destroy infectious organisms [7]. Antibodies (immunoglobulins) are a key humoral immune protein with multiple defensive functions [7,8]. The antibody proteins are produced by B-lymphocytes, and they bind to foreign molecules to induce numerous defensive effects, including neutralization of pathogen function, labeling of pathogens for attack by defensive cells, and facilitating defensive molecule binding to pathogens (e.g., complement proteins) [7,9].

Natural antibodies (NAbs) are a sub-category of immunoglobulins that are part of the innate immune response [10,11,12]. They are expressed prior to immune challenge or infection and provide protection while the adaptative immune system develops; therefore, they are beneficial in the protection against infection, and they represent plausible indicators to determine susceptibility/resistance [7,10,11,12,13]. Importantly, NAbs are thought to facilitate the adaptive immune response by supporting pathogen recognition, tagging (opsonization), and antigen presentation [14,15,16,17,18]. Adaptive antibodies (AAbs) are the best-studied immunoglobins. These molecules are produced following a pathogen encounter, through a series of regulatory steps that include pathogen phagocytosis, antigen presentation, regulatory T-lymphocyte signaling, and stimulation of B-lymphocytes to produce antigen-specific antibodies with high specificity [7]. This reflects the adaptive immune response with increased specificity and effectiveness of immune defense. Adaptive antibodies are the target of vaccination, with the goal to stimulate the production of AAbs specific to select pathogens prior to any infection. 

Animals are known to differ in susceptibility to diseases, and a part of this variation can be explained by host genetics [19,20,21]. Antibody levels are known to be controlled by multiple genes [22]; therefore, selection may improve antibody-mediated immunity [23]. The direct measurement of disease resistance phenotypes is costly and requires long-term experimental challenges or large and accurate data collection in the field for endemic diseases. Fulton et al. [24] showed that selection against Marek’s disease-induced mortality can be effective if performed consistently over extended periods of time. However, disease resistance phenotypes are not available on breeding stocks kept in bio-secure environments; therefore, alternative proxy traits should be explored to enable genetic selection for disease resistance. Genetic variation exists for antibody response after vaccination; however, the literature estimates of heritability are low to moderate, with values ranging from 0.04 to 0.31 depending on the population, method of estimation, and time span after vaccination [25,26,27,28,29]. In a study including IBD, IBV, and NDV following the challenge and using a variety of methods, the estimates of heritability ranged from 0.0 to 0.58 for IBD, 0.0 to 0.58 for IBV, and 0.0 to 0.37 for NDV [30].

Natural antibody levels have been shown to have a genetic component. The literature estimates of heritability are not abundant [29,31,32,33]; some studies focusing on anti-KLH responses have found values of between 0.05 and 0.28 for the general response or for specific immunoglobulins (IgT, IgM, IgA, and IgG), indicating a range of values similar to the ones reported for response to vaccination above. ivergent selection for low or high Nab levels to keyhole limpet hemocyanin (KLH) resulted in lines with differential responses [34]. Similar divergently selected populations have been studied to gain insights into the genetics of antibody response and to identify QTL regions [35,36] and candidate genes [37] that impact the immune response. A candidate gene (TLR1A) with a strong effect on the level of IgM was identified in a White Leghorn population [38]. The studies were, however, limited in genomic resolution to a 60 k SNP chip and were also limited to a single artificially created or existing chicken line. Such results should be confirmed across commercially used lines for the possible implementation in breeding programs designed to produce commercial birds. It must also be noted that the high antibody response to SRBC line was shown to result in more resistance for some but not all disease challenges [23]. Therefore, it may be necessary to consider more than a single antigen to achieve better overall disease resistance. Wondmeneh et al. [39] showed opposing effects of increased antibody levels on survival in two layer lines (positive in ISA Brown but negative in the Ethiopian Horo breed), which suggests that the effects may be line-dependent. To overcome these limitations, the objective of this study was to analyze the genetic background of natural antibodies binding KLH, OVA, and PHA and adaptive antibodies binding IBD, IBV, NDV, and REO in eight lines of elite layer chickens, which represent three breeds used for commercial egg production, White Leghorn (WL), Rhode Island Red (RIR), and White Plymouth Rock (WPR). To the best of our knowledge, no previous research paper has evaluated the genetic basis for multiple NAbs and AAbs in the same animals, which will make the study herein unique in the chicken immunogenetic literature. The genotype information was obtained from low pass sequences (1× and 4×) and from 54 K Axiom SNP arrays.

## 2. Materials and Methods

### 2.1. Animals and Phenotypes

In an initial study (manuscript in progress), antibody responses to vaccination were measured for common chicken viral diseases (Infectious bursal disease (IBD), Infectious Bronchitis Virus (lBV), Newcastle Disease Virus (NDV), and Reovirus (REO)). Additionally, we assessed the level of NAbs binding keyhole limpet hemocyanin (KLH), Ovalbumin (OVA), and phytohemagglutinin (PHA) for those same samples. Antigens such as KLH (a protein extracted from the sea snail Megathura crenulate) and PHA (a protein from the red kidney bean Phaseolus vulgaris) are used to measure nAb as poultry are not commonly exposed to them, while OVA is a protein of the egg and thus is an autoantigen. The vaccination protocol was a typical commercial vaccination program with birds being vaccinated 2–4 times for each antigen between day of hatch to 14 weeks of age. All birds received a 4-way vaccine for the four aforementioned viral diseases at 14 weeks of age. A total of 4446 plasma samples from males and females representing four generations (2017J to 2019M) from eight lines were used. These represented lines from three breeds used in commercial layer production: four White Leghorn lines (WL1 to WL4), two White Plymouth Rock lines (WPR1 and WPR2), and two Rhode Island Red lines (RIR1 and RIR2) from Hy-Line international (P.O. Box 310, Dallas Center, IA 50063, USA). 

### 2.2. Antibody Levels Using ELISA

Plasma samples of 45–50 week-old birds were obtained from blood that was collected into tubes containing EDTA. Blood samples were either fresh, or previously spun at 1000 g prior to freezing. Each plasma sample was split into two tubes, with one tube set being shipped to Washington State University for testing for natural antibodies (anti-KLH, anti-OVA, and anti-PHA) and the other set being retained at Hy-Line for testing for adaptive antibodies (anti-IBD, anti-IBV, anti-NDV, and anti-REO). Antibody levels were detected via ELISA (enzyme-linked immunosorbent assay). The adaptive antibodies against IBD, IBV, NDV, and REO were measured using kits from BioChek (Scarborough, ME, USA). The manufacturer’s recommended protocol was followed, with plasma diluted to 1/500 and Sample to Positive control ratios (S:P) and titers determined using the BioChek software ver. 2020. The ELISA protocol for the NAbs measured IgY antibodies binding KLH, PHA, and OVA using the single-point sandwich ELISA method as described in [40]. For the present study, plasma from males and females from additional generations were utilized. Plasma samples were analyzed in triplicate and expressed as SP ratios (0 to 1). A ratio of 0 indicates an antibody level equal to that of the negative control provided with the diagnostic kit, while a value of 1 indicates an antibody level equal to that of the positive control provided with the diagnostic kit. After confirmation of high correlation between the three replicates, records within a sample were averaged.

### 2.3. Genotyping Using the 54 K Axiom^TM^ SNP Chips and Low-Pass (4×) Sequencing

For estimation of heritability and adjustment for population structure, SNP genotypes were obtained for each sample using a proprietary 54 K Affymetrix Axiom SNP (ThermoFisher, Santa Clara, CA, USA). These SNP are a subset of SNP from the Axiom 600 K chicken SNP array. Genotyping was performed at GeneSeek (Lincoln, NE, USA). Affymetrix analysis power tool was used for genotype calling.

For GWAS, individual DNA genome sequences were obtained at either 4× or 1× coverage. Libraries were prepared with 0.137 ng of DNA using Illumina Nextera XT kit (Illumina, San Diego, CA, USA). Twenty-four individually bar-coded libraries were then pooled and sequenced together on one lane of a HiSeq X with 2 × 150 bp reads. Imputations to genome sequence were provided by GenCove (New York, NY, USA) [41]. The number of used SNPs after filtering for MAF > 0.01 were 9,361,049, 12,225,322, and 10,948,752 for RIR, WL, and WPR, respectively.

### 2.4. Data Analysis

Differences between lines, generations, and sexes were assessed using lm() function in R ver 4.2.2 [42] while separating data into subsets to avoid complete confounding for the factors. After an initial screening with a subset of samples (100 males per line in a single generation), additional plasma samples were evaluated for males and females from four consecutive generations to increase the power of the experiment for most promising line–antigen combinations, and to enable genetic and genomic analyses. The dataset used is summarized in Table 1. Heritability was estimated using pedigree and genomic relationship matrices based on genotypes. A linear mixed model with equation

Y = mu + genlinesex + SNP + G + e

was fitted, including the fixed effect of the overall mean (mu) and the combined effect of generation–line–sex (genlinesex) and SNP. The random effects of animal with G matrix (fitted in ASReml [43] and GCTA [44]), and residual. Each line was analyzed separately in the pedigree model except the two Rhode Island Red lines that had some common ancestors and were analyzed jointly. For the genomic analysis, lines of the same breed were also combined to increase the power of the GWAS. Low-pass sequencing (4× and 1×) was used to obtain genomic data for GWAS. QQplots (Supplementary Materials) were created using library (qqman) in R [45]. Genomic inflation factors ranged from 0.414 to 1.002, suggesting lack of unaccounted population stratification. The SNPs with *p*-values below 10^−6^ were considered significant and used to identify potential candidate genes. Using Bonferroni correction with number of SNPs (9 to 12 M above) would be much too stringent; using unadjusted 0.01 would allow too many false positives, so 10^−6^ is a compromise between these two approaches. Functional annotation of significant SNPs was performed in SNPEff [46] using GRCGalGal6 genome build (https://www.ncbi.nlm.nih.gov/assembly/GCF_000002315.5/ accessed on 8 May 2024). Enrichment and pathway analyses were conducted in Panther [47], with focus on genes with significant SNPs classified as having moderate or high impact, and those on immune-related pathways, and it was carried out for combined antigens and separately for Nabs and Aabs.

## 3. Results

The overall summary of basic trait statistics for antibody levels (SP ratios) by line, sex, and generation is shown in Table 1. Analyses were carried out for each antigen and line separately. The genetic lines used in the study herein represent samples of the distinct breeds available for egg commercial production; therefore, the results are relevant to the egg industry. In a preliminary analysis, the effects of line, generation and sex were significant to explain variation in antibody expression. In general, means for antibody expression were similar across lines by antigen. Antibody expression after vaccination against common viral diseases tended to be higher than those for natural antibodies. Average values (across lines) for Aabs were 3.02, 1.97, 4.94, and 1.43 for IBD, IBV, NDV and REO, respectively; 0.91 and 0.62 for Nabs KLH and PHA; and 1.27 for OVA, an egg albumen autoantigen. Antibody values were, in general, different between sexes for all antigens and varied across generations.

### 3.1. Response to Vaccination against Common Viral Diseases

#### 3.1.1. Response to Vaccination against Infectious Bursal Disease Virus (IBD)

Sample numbers were sparse in generations 2017J and 2018K, but they were better balanced across lines in the last two generations (2028L and 2019M). Female samples were available only in one WL line in one generation (2019M), but mean antibody levels were similar in both sexes (F = 5.1 and M = 5.6). Significant differences in antibody levels were found between generations and lines for IBD. Average antibody levels across lines (3.02) were in good agreement with the corresponding within-breed values of 3.10, 2.64 and 2.75 for RIR, WL and WPR, respectively. On average, the antibody levels ranged between 1.76 (WL3) and 3.64 (WL4), and there was a clear and consistent ranking of antibody levels among the lines. The antibody levels significantly increased with generation from 1.96 to 3.65 after accounting for generation-line in the model. The Supplementary Materials include a boxplot distribution of antibodies against IBD for each generation and line–sex combination (Figure S1). 

Estimates of variance components for IBD antibody responses are shown in Table 2. Pedigree estimates of heritability ranged from 0.00 (RIR1) to 0.30 (WL4) and tended to be greater for white-egg lines (WL) than for brown-egg lines (WPR and RIR). Genomic estimates were similar to pedigree-based ones but not for all lines. The average estimates of heritability across lines were 0.10 (pedigree) and 0.07 (genomic). For lines WL1, WL3, WL4 and WPR2 estimates of heritability using both pedigree and genomic relationship matrices were larger than their standard errors. Genomic heritability was significantly greater than zero for WL4. 

**Figure 1:**
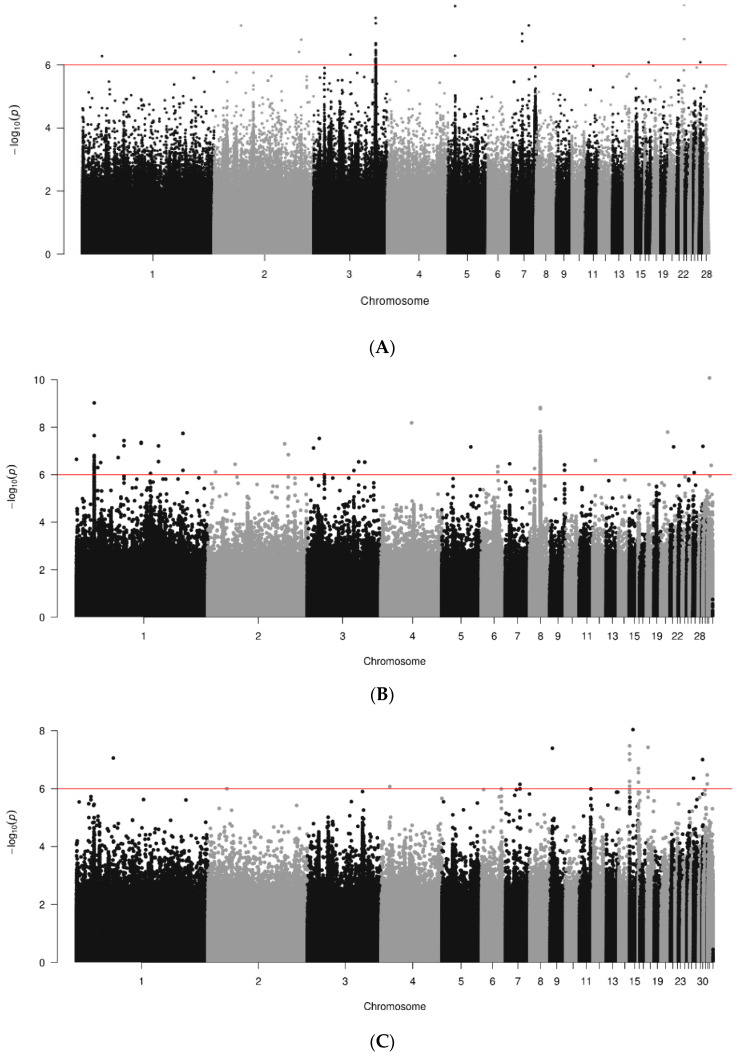
Results of GWAS for antibody levels against IBD by breed ((**A**). RIR; (**B**). WL; (**C**). WPR). Red line marks 10^−6^ significance level.

Results of GWAS, in the form of Manhattan plots, are shown for data joined by breed in Figure 1. A total of 400, 785 and 398 genome sequences were analyzed for RIR, WL and WPR lines, respectively. Several significant SNPs (*p* < 10^−6^) were found across the genome, but they tended to be breed specific. More significant regions were found in the macro chromosomes for WL than for RIR and WPR lines. In white-egg lines (Figure 1B) there were significant SNPs identified on chr. 1 to 10, 12, 20, 21, 27, 30 and 33, with the strongest one in chr. 20 (1.60092 × 10^−8^; bp 10251058). RIR lines (Figure 1A) showing significant regions in chr. 1, 2, 3, 5, 7, 17, 22 and 27; max signal in chr. 22 (1.2168 × 10^−8^; bp 4621636). WPR lines (Figure 1C) showed significant SNP in chr. 1, 4, 7, 9, 14, 15, 16, 18, 27, 30 and 31; max signal in chr. 22 (3.74019 × 10^−8^; 1899993 bp).

#### 3.1.2. Response to Vaccination against Infectious Bronchitis Virus (IBV)

Significant differences in antibody levels were found between generations and lines for IBV (See Figure S2 in the Supplementary Materials). Line average antibody levels ranged between 0.76 (WL3) and 3.08 (WPR2). Average antibody levels across lines (1.97) were in good agreement with the corresponding within breed values of 1.56, 1.59 and 2.76 for RIR, WL and WPR, respectively. On average, White Plymouth Rock lines tended to have a stronger response than RIR and WL lines. Antibody values tended to increase significantly with generation from 1.44 to 2.13 after accounting for generation–line in the model. Female samples were available only for one WL line in one generation (2018L), but mean SP ratios were not significantly different between sexes (F = 0.78 and M = 1.08). 

Estimates of variance components for antibody responses against IBV are shown in Table 3. Pedigree estimates of heritability ranged from 0.00 (WL14, WPR1) to 0.58 (WL3). Genomic estimates were similar to pedigree-based estimates. The average estimates of heritability across the lines were 0.23 (pedigree) and 0.15 (genomic). At least one line for each breed showed intermediate heritability for both pedigree and genomic-based values. Lines RIR2 and joined, WL3 and WPR2 had estimates of genomic significantly greater than zero. 

**Figure 2:**
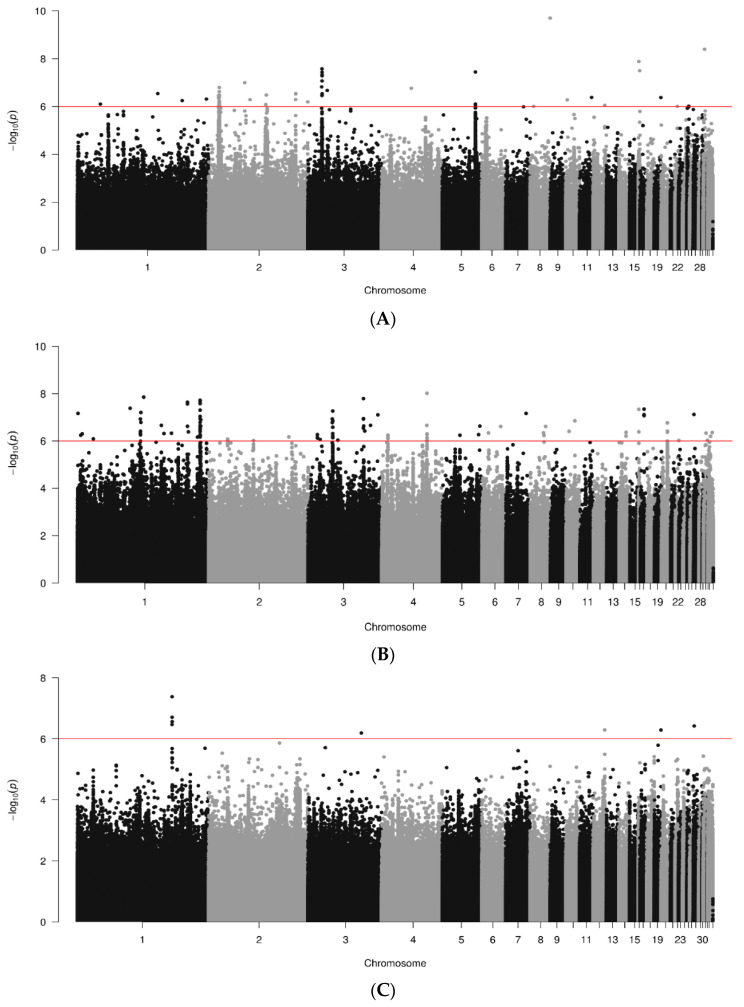
Results of GWAS for antibody levels against IBV by breed ((**A**). RIR; (**B**). WL; (**C**). WPR). Red line marks 10^−6^ significance level.

Manhattan plots of GWAS for antibody responses against IBV are shown for data joined by breed in Figure 2. A total of 497, 582, and 299 sequences were analyzed for the RIR, WL and WPR lines, respectively. Several significant SNPs (*p* < 10^−6^) were found across the genome, but they tended to be breed-specific, and more were present in the RIR and WL lines (Figure 2A,B) than in the WPR lines (Figure 2C). For the RIR lines, significant SNPs were found in chr. 1 to 5, 8, 10, 11, 12, 16, 19, 22, 25, and 31; max signal in chr. 8 (2.000 × 10^−10^; bp 30190326). White-egg (WL) lines had several significant SNPs found on chr. 1 to 8, 10, 14, 16, 17, 20, 22, 27, 31, and 33, with the strongest one at chr. 2 (2.43273 × 10^−11^; bp 81136672). The WPR lines showed a few significant SNPS in chr. 1, 3, 12, 19 and 27, with the highest signal on chr. 22 (4.15083 × 10^−8^; bp 142467636).

#### 3.1.3. Response to Vaccination against Newcastle Disease Virus (NDV)

Significant differences in antibody levels were found between generations, lines and sexes for response to NDV (see Figure S3, Supplementary Materials). Line average antibody levels ranged between 3.24 (WL3) and 5.38 (WPR2). The average antibody levels across the lines were 4.94, and the corresponding within-breed values were 5.93, 4.14, and 5.56 for RIR, WL, and WPR, respectively. Brown-egg lines (RIR and WPR) tended to have a slightly stronger response than the white-egg counterparts (WL). Antibody values tended to increase significantly with generation from 3.21 to 5.71. Female samples were available only in one WL line in one generation (2018L); the SP ratios for females (5.82) were significantly greater than the ones for males (4.41). 

Estimates of variance components for antibody responses against NDV are shown in Table 4. Pedigree estimates of heritability ranged from 0.00 (RIR1, WL4) to 0.55 (WPR1). Genomic estimates (0.00 to 0.38 for the same lines) tended to be slightly lower than the pedigree ones. The average estimates of heritability across lines were 0.17 (pedigree) and 0.10 (genomic). A few lines had estimates of heritability that were greater than the corresponding standard error (RIR2, WL1-3, and WPR1), and two of them (WL3 and WPR1) had intermediate to high values. 

**Figure 3:**
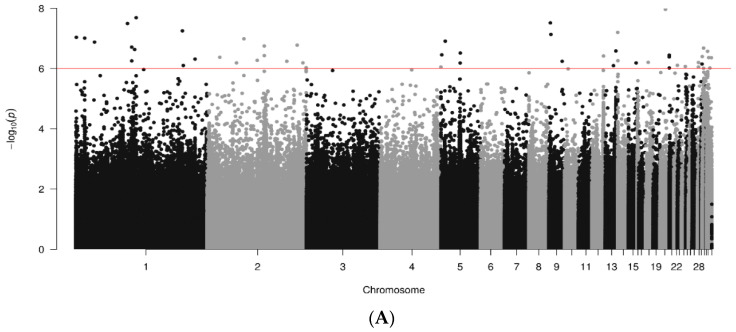

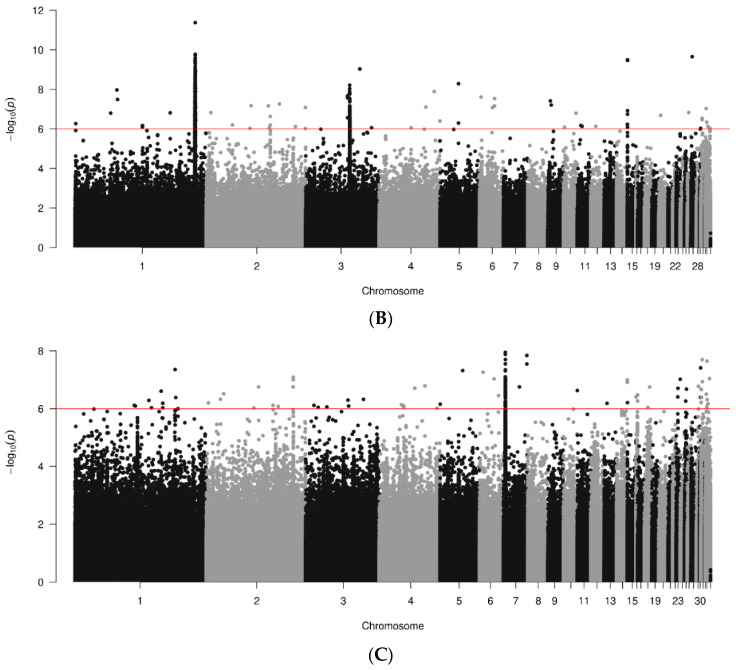
Results of GWAS for antibody levels against NDV by breed ((**A**). RIR; (**B**). WL; (**C**). WPR). Red line marks 10^−6^ significance level.

Whole genome association results represented as Manhattan plots for NDV antibody responses are shown for data joined by breed in Figure 3. A total of 400, 737 and 300 genome sequences were analyzed for RIR, WL and WPR lines, respectively. Several chromosomes shown significant SNPs (*p* < 10^−6^) for the three breeds. For RIR lines (Figure 3A) significant regions were found in chr. 1, 2, 4, 5, 9, 12, 13, 14, 15, 18, 20, 21, 22, 24, 28, 30, 31 and 33; max signal in chr. 20 (1.08338 × 10^−8^; bp 8175379). White-egg (WL) lines (Figure 3B) had several significant SNPs ranging chr. 1 to 6, 9, 10, 11,12, 15, 18, 20, 26, 27, 30, 31 and 33; two regions in chromosomes 1 and 3 were represented by a sizeable number of significant SNPs in clusters. The strongest SNP, however, was in chr. 18 (1.17167 × 10^−13^; bp 8011255). WPR lines (Figure 3C) showed significant regions in chr. 1 to 7, 8, 11, 14, 16, 18, 23, 25, 30, 31 and 33; with maximum signal in chr. 16 (1.97348 × 10^−9^; bp 2612501).

#### 3.1.4. Response to Vaccination against Reovirus (REO)

Significant differences in antibody levels were found between generations, lines and sexes for REO (see Figure S4, Supplementary Materials). Line average antibody levels ranged between 1.03 (WL3) and 1.60 (WPR2). Average antibody levels across lines (1.43) were in good agreement with the corresponding within breed values of 1.53, 1.27 and 1.61 for RIR, WL and WPR, respectively. Brown-egg lines (RIR and WPR) tended to have a slightly stronger response than the white-egg lines (WL) for NDV. Antibody values tended to increase significantly with generation from 1.05 to 1.70. Female samples were available only in one WL line in one generation (2018L); the SP ratios for males (1.38) were significantly greater than the ones for females (0.76). 

Estimates of variance components and heritability for antibody responses against REO are shown in Table 5. Pedigree estimates of heritability ranged from 0.00 (WPR lines and WL4) to 0.42 (WL1, 3). Genomic estimates ranged from 0.00 (WPR lines and WL1, 2, and 4) to 0.22 (RIR2 WL3) and tended to be slightly lower than the pedigree estimated values. The average estimates of heritability across lines were 0.14 (pedigree) and 0.06 (genomic). Two analyses (RIR2 and RIR-joined) had genomic estimates of heritability that were greater than the corresponding standard error, and these had intermediate values.

**Figure 4:**
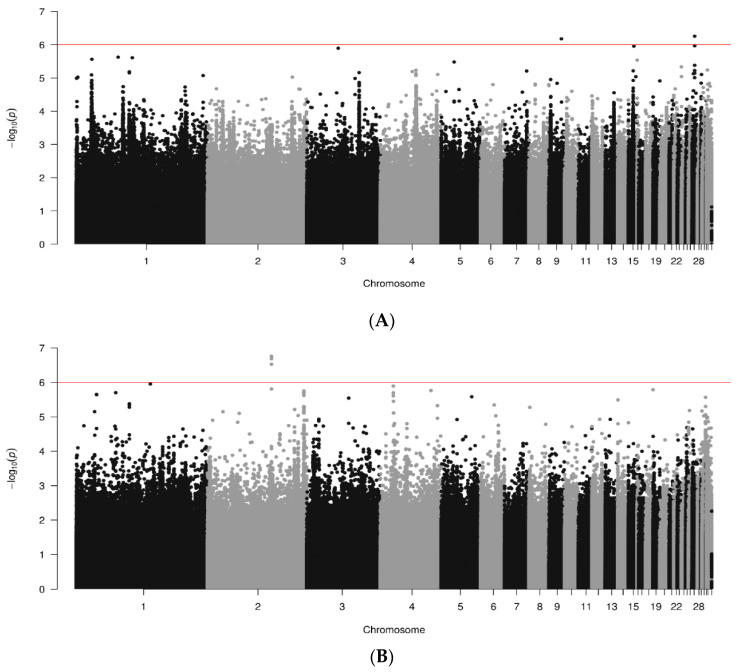

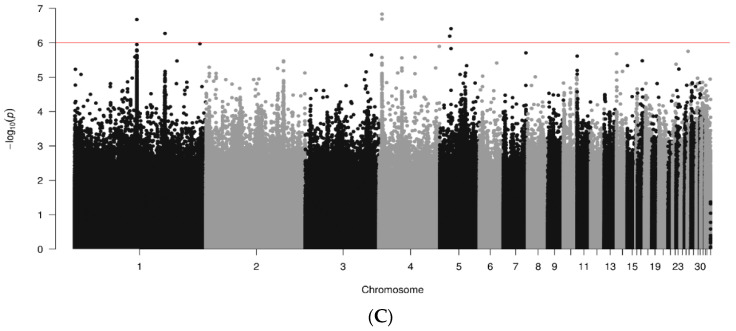
Results of GWAS for antibody levels against REO by breed ((**A**). RIR; (**B**). WL; (**C**). WPR). Red line marks 10^−6^ significance level.

Whole genome association results represented in the form of Manhattan plots for REO antibody responses are shown for data joined by breed in Figure 4. A total of 448, 487 and 300 sequences were analyzed for RIR, WL and WPR lines, respectively. Only a few chromosome regions showed significant SNPs (*p* < 10^−6^) for the three breeds. For RIR lines (Figure 4A) one SNP in chr. 9 and one in chr. 7 showed significant but weak response (i.e., 10^−7^). For white-egg (WL) lines (Figure 4B) three SNPs had significant but again low responses in chr. 2. WPR lines had significant regions in chr. 1, 4 and 5, with two SNPs each, but they were also weak (Figure 4C).

### 3.2. Natural Antibodies

For NAb levels specific for foreign antigens (KLH and PHA) and an autoantigen (OVA), only data for males in three generations (2017J to 2018L) were available. In general, Nab responses against external antigens were weaker than the Aabs after vaccination against common viral vaccines (on average across line the antibody levels for KLH and PHS were of 0.91 and 0.62 vs. 2.84 across viral diseases). The response for the autoantigen (OVA) was intermediate (on average across lines of 1.27).

#### 3.2.1. Natural Antibodies for Keyhole Limpet Hemocyanin (KLH)

Sample numbers were sparse in generations 2017J and 2018K, but they were better balanced in the last generation (2028L). Significant differences in anti-KLH antibody levels were found between lines, but not for generation (see Figure S5, Supplementary Materials). Line average antibody levels ranged between 0.64 (WL3) and 1.20 (WPR1). Average antibody levels across lines (0.91) were in relatively good agreement with the corresponding within breed values of 0.88, 0.74 and 1.18 for RIR, WL and WPR, respectively. Higher antibody expression was observed for WPR lines than for the other lines. The antibody levels significantly increased with generation from 3.21 to 5.71.

Estimates of variance components and heritability for anti-KLH antibody levels are shown in Table 6. Estimates of heritability (pedigree and genomic) approached zero for WL lines. Average across lines values were similar with 0.15 for pedigree and 0.16 for genomics estimates. However, they tend to be relatively erratic for RIR and WPR lines. They were high and significantly different from zero for RIR2 and WPR2; but, when a joined genomic analysis was carried out, the heritability estimates for these lines were 0.25 and 0.14, respectively. 

**Figure 5:**
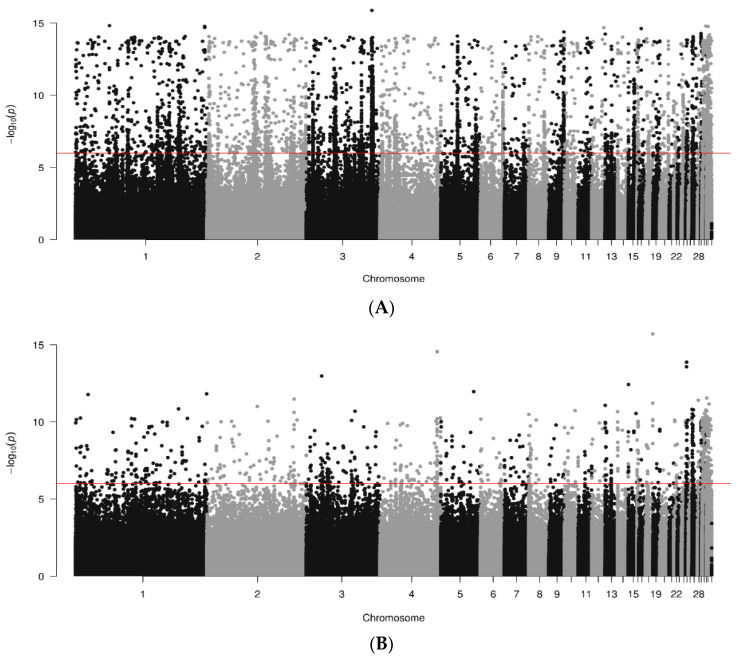

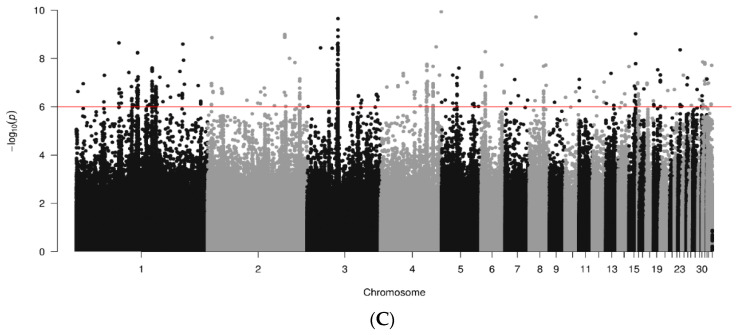
Results of GWAS for antibody levels against KLH by breed ((**A**). RIR; (**B**). WL; (**C**). WPR). Red line marks 10^−6^ significance level.

Results of GWAS for anti-KLH antibody levels are shown for data joined by breed in Figure 5. A total of 198, 389 and 197 sequences were analyzed for RIR, WL and WPR lines, respectively. Many more significant SNPs and regions were found for natural antibody levels against this antigen than for any of the other antigens tested. Many significant chromosomal regions and SNPs (*p* < 10^−6^) were found across the genome for the three breeds. For RIR lines significant SNPs were found at each chromosome, except for chr. 29. There were 5046 significant SNPs across the genome. The maximum number of signals were in chr. 3 (two adjacent SNPs at bp 98467405 and 9846740; 1.30 × 10^−16^. The estimated effects for these two SNPs were 2.78 ± 0.335) and chr. 1 (one SNP at bp 50854746; 1.48 × 10^−15^. The solution for this SNP was 2.89 ± 0.362). However, there were 37 other SNPs with high *p*-values (i.e., <10^−15^) in chr. 1, 2, 3, 4, 5, 8, 9, 12, 13, 14, 17, 27 30, 31, and 33. White-egg (WL) lines had a total of 1064 significant SNPs spread across all chromosomes, with the strongest one at chr. 18 (−6.32 × 10^−16^; bp 10993468) There were three other highly significant SNPs (*p* < 3 × 10^−15^) in chr. 4 (bp 85884362 to 85884367; solutions 2.167). WPR also showed a sizeable number of significant regions and SNPs in all chromosomes, except for chr. 21 and 29, with a total of 507 significant SNPs across the genome. The four largest signals (*p* < 6.5 × 10^−10^) were in chromosomes 4, 8, 3, and 15. The strongest one in chr. 4 (1.16 × 10^−10^; bp 91000625) had an SP ratio of 1.943.

#### 3.2.2. Natural Antibodies Binding Ovalbumin (OVA) 

Sample numbers were sparse in generation 2017J and 2018K, but they were better balanced in the last generation (2018L). Significant differences in anti-OVA antibody levels were found between lines, but not generation (see Figure S6, Supplementary Materials). Line values ranged from 0.27 (WL2) to 1.171 (WPR2). Average antibody levels across lines were 1.27, with corresponding within-breed values of 1.50, 0.76, and 1.98 for RIR, WL and WPR, respectively. WL lines had significantly lower antibody levels than RIR and WPR lines. The WPR lines had numerically higher antibody levels than RIR lines, but this difference did not reach statistical significance. 

Estimates of variance components and heritability for anti-OVA antibody levels are shown in Table 7. Pedigree estimates of heritability approached zero for WL and WPR1 lines. The same was the case for genomic estimates, except for WL2 (0.24). On average across lines, they were similar, with values of 0.15 (pedigree) and 0.15 (genomics). However, only one line (RIR2) had an estimate that was significantly different from zero. Lines RIR2 and WL4 had heritability estimates that were larger than their standard errors. 

**Figure 6:**
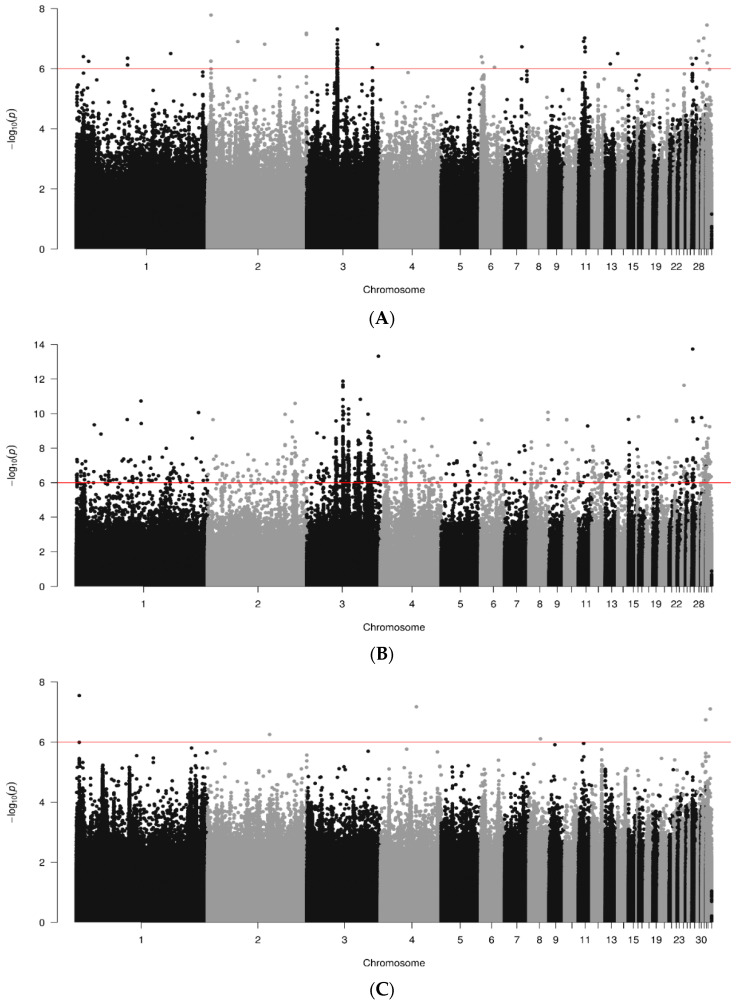
Results of GWAS for antibody levels against OVA by breed ((**A**). RIR; (**B**). WL; (**C**). WPR). Red line marks 10^−6^ significance level.

Whole genome association results represented in the form of Manhattan plots for OVA autoantibody levels are shown for data joined by breed in Figure 6. A total of 198, 389 and 197 sequences were analyzed for RIR, WL and WPR lines, respectively. Several significant (*p* < 10^−6^) chromosomal regions were detected, mainly for RIR and WL breeds. For RIR lines (Figure 6A) 57 significant SNPs were found in chromosomes 1, 2, 3, 6, 7, 11, 13, 26, 27, 28, 31 and 33. The strongest signal was in chr. 2 (1.642 × 10^−8^; bp 5172770). The WL lines (Figure 6B) had several signals, including 1142 SNPs spanning all chromosomes, except chr. 21 and 29. The two strongest SNPs (*p* < 5 × 10^−14^) were in chr. 27 and 3. The one in chr. 27 (1.823 × 10^−14^; bp 975460) had an estimated effect on antibody level of 2.61. The one in chr. 3 (4.76 × 10^−14^; bp 107701095) had an SP ratio solution of 2.22. The WPR lines (Figure 6C) only had six significant regions in chromosomes 1, 2, 4, 8, 31, and 33, with the strongest SNP in chr. 1 (2.85 × 10^−14^; bp 4320170). 

#### 3.2.3. Natural Antibodies Binding Phytohemagglutinin (PHA)

Sample numbers were sparse in generations 2017J and 2018K, but they were better balanced in the last generation (2018L). Significant differences in antibody levels were found between lines, but generation was not significant in explaining variation for this trait (see Figure S7, Supplementary Materials). Average antibody levels across lines (0.62) were in good agreement with the corresponding within breed values of 0.62, 0.59 and 0.58 for RIR, WL and WPR, respectively. Average line values ranged from 0.55 (WL3) to 0.81 (WL4). 

Estimates of variance components and heritability for anti-PHA antibody levels are shown in Table 8. Pedigree estimates of heritability (pedigree and genomic) approached zero for RIR1, WL1, 3, 4 and WPR1 lines. On average across lines, they were similar, with values of 0.06 (pedigree) and 0.08 (genomics). Estimates of heritability were no different from zero for all lines due to large standard errors; but, the genomic estimate for RIR1 (0.46) was greater than its standard error. 

**Figure 7:**
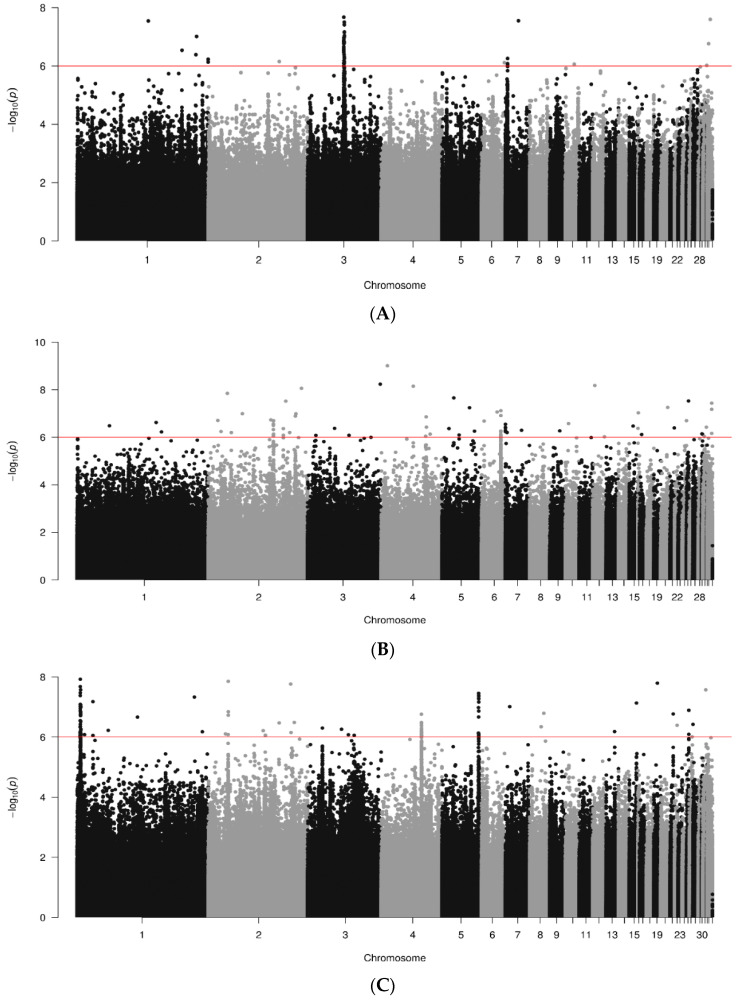
Results of GWAS for antibody levels against PHA by breed ((**A**). RIR; (**B**). WL; (**C**). WPR). Red line marks 10^−6^ significance level.

Manhattan plots describing the WGA association results for anti-PHA antibody levels are shown for data joined by breed in Figure 7. A total of 198, 389 and 197 genomic sequences were analyzed for the RIR, WL and WPR lines, respectively. Several significant (*p* < 10^−6^) chromosomal regions were detected, mainly for breeds WL and WPR. For RIR lines (Figure 7A) only 51 significant SNPs were found in chromosomes 1, 2, 3, 6, 7, 10, 31 and 33. The strongest signal was in chr. 3 (top SNP 3.01 × 10^−9^; bp 56189978), supported by several SNP in the vicinity. The WL lines (Figure 7B) had a few more significant regions than the RIR, including 81 SNPs on chromosomes 1 to 7, 9, 10. 12, 15, 16, 17, 20, 21, 25, 30, 31 and 33, including the strongest SNPs (*p* < 9.78 × 10^−10^, bp 9282595). For WPR lines (Figure 7C), more significant SNPs were found than for the other two breeds, with a total of 113 spanning chromosomes 1 to 5, 7, 8, 13, 15, 19, 21 25, 26, 27 and 31, with the strongest SNP in chr. 1 (1.19 × 10^−8^; bp 4705323).

### 3.3. Pathway Enrichment

The SNPs with *p*-values below 10^−6^ were annotated using SNPEff [46]. The gene set that contained the SNPs was analyzed for pathway enrichment in PANTHER [47]. A summary of the top results of gene enrichment is listed in Table 9 for the joined analysis (top) and for the Nabs (bottom) of Rhode Island Red lines. Interestingly, for SNP found in the joined antibody analysis, the most significant enrichment was found for categories such as defense/immunity protein, immunoglobulin receptor superfamily, and antimicrobial response protein. The corresponding results for the natural antibody analysis found the highest overrepresentation in the immunoglobulin receptor superfamily and the defense/immunity proteins. Table 10 shows similar results for the joined and split analysis in the White Leghorn lines. Enrichment genes for White Leghorn lines were for the categories immunoglobulin receptor superfamily, defense/immunity protein (both pooled and split analyses), and protein modifying enzyme (joined analysis), and zinc finger transcription factors (natural antibody analysis). The White Plymouth Rock joined analysis did not show significant overrepresentation, but some of the identified SNPs were annotated to immune-relevant pathways. This breed, however, showed some significant overrepresentation of zinc finger transcription factors, as summarized in Table 11. The corresponding list of PANTHER mapped genes, including gene ID, name, symbol, persistent id, orthologs, PANTHER family/sub-family, and protein class, are presented in Addendum 1 for joined analyses by breed. 

## 4. Discussion

In the study herein, we evaluated the genetic basis for innate (natural) and induced (adaptive) antibodies in chickens using elite egg production lines from three chicken breeds (Rhode Island Red, White Leghorn, and White Plymouth Rock). Natural antibodies were measured using enzyme-linked immunosorbent assays with external antigens (KLH and PHA) and an autoantigen (OVA). Adaptive antibody responses were measured after vaccination for common chicken viral diseases (IBD, IBV, NVD, REO) using commercial ELISA kits. Data utilized for this study included eight lines representing three breeds (RIR, WL and WPR) and multiple generations (Table 1). For some generation–line combinations both male and female information was available. Levels of antibodies in vaccinated birds against viral diseases were variable across generations, lines and, in some cases, sexes. Antibody responses after vaccination against common viral diseases tended to be higher than those for natural antibodies. Average values (across lines) for the vaccines were 3.02, 1.97, 4.94, and 1.43 for IBD, IBV, NDV, and REO, respectively; 0.91 and 0.62 for naive antigens for chickens KLH and PHA; and 1.27 for OVA, an egg albumen autoantigen. 

The principles of vaccination and the mechanisms that they induce to promote immune responses are well-known; for a general review, see [48]. The use of vaccines in poultry production is well-established [49]; for a review of the specific vaccines against poultry viruses of global and commercial importance, including the ones studied herein, see [50]. The antibody response to vaccination in chickens has been well-studied (e.g., [30,51]). In our study, there were significant differences in antibody responses to each antigen among the lines. The brown-egg lines tended to have greater antibody responses against IBD (WPR), IBV (WPR), NVD (WPR) and REO (RIR, WPR) antibodies than the WL (white-egg) lines. This could be related to higher levels of genetic variation in immune related genes in brown-egg lines compared to WL as indicated by higher number of haplotypes observed for the chicken Major Histocompability Complex (MHC) (unpublished). The magnitude of the heritability estimates was quite erratic, and no general conclusion could be reached. Estimates ranged from zero to low to high depending on the line and vaccine (Table 2, Table 3, Table 4 and Table 5). This can be in part due to the data structure and the limited information contained in the data to properly estimate genetic parameters, as was evident from the large standard errors associated with heritability estimates for all disease cases. However, if we consider the average values across lines, the pedigree heritability estimates were 0.10, 0.22, 0.17 and 0.14 for antibodies against IBD, IBV, NDV and REO, respectively. The corresponding genomic estimates were 0.07, 0.15, 0.10 and 0.06, respectively. These results indicate that selecting for antibody responses to vaccination must be evaluated on a case-by-case basis. Literature estimates of heritability for antibody response after vaccination support low to moderate values and a wide range of values ranging from 0.04 to 0.31 depending on the population, method of estimation, and time span after vaccination [25,26,27,28,29]. In a study including IBD, IBV, and NDV following challenge and using a variety of methods, estimates of heritability ranged from 0.0 to 0.58 for IBD, 0.0 to 0.58 for IBV and 0.0 to 0.37 for NDV [30]. Our results were more consistent showing the presence of more between-population genetic variation than within-line (estimates of heritability) genetic variation for vaccine response, which may impact the design of breeding strategies targeting this trait.

Natural antibodies present with no known prior contact with a specific antigen are also studied in chickens. Parmentier et al. [31] observed variations in anti-KLH and anti-OVA antibodies in chickens divergently selected for antibody response. Berghof et al. [33] used chicks of the high and low NAb response lines and confirmed that selective breeding for high KLH-binding levels increased pathogen resistance. We detected significant differences among lines in levels of NAb specific for KLH, OVA and PHA, and found that brown-egg lines (WPR and RIR) showed higher anti-KLH antibody levels than the white-egg (WL) lines, as was the case for the adaptive antibody response after vaccination. This is not a generalized result. It is well-known that brown-egg type layers have higher MHC polymorphism than white-egg layers, and this could also contribute to a more robust immune defense. Response to vaccination can vary depending on many factors, including the route of administration. A recent study compared different vaccination regimens and chicken breeds in three trials [52]. The study showed varying immune response levels. For instance, Barred Rock layers and Rhode Island Red pullets showed a strong immune response in both serum and egg yolk when vaccinated intramuscularly and subcutaneously with a KLH–enterobactin conjugate. However, the opposite occurred following intradermic application, for which White Leghorn showed a stronger response. So, as in the case of responses to vaccination, some genetic variation in Nabs is present in commercial layer populations. The magnitude of heritability estimates for Nab response herein was also variable and no solid conclusion could be reached. Estimates ranged from zero to low to high depending on the line and antigen (Table 6, Table 7 and Table 8). Several estimates were close to the lowest boundary of the parameter space and may indicate that not enough information was contained in the data for some antigen-line combinations. As for the case of vaccine responses, there were large standard errors associated with heritability estimates for NAb levels. However, if we consider the average values across lines, the pedigree heritability estimates were 0.21, 0.15 and 0.06 for antibodies anti-KLH, anti-OVA and anti-PHA, respectively. The corresponding genomic estimates were 0.16, 0.15 and 0.08, respectively. So overall, these results indicate that these traits are relatively heritable and can be incorporated in a selection strategy to improve general immunity; however, implementation must be evaluated for each population and antigen. Published research showing estimates of heritability for NAb expression is not extensive; however, a few papers show results that indicate relatively low to intermediate values. Berghof et al. [32] found estimates ranging from 0.05 to 0.17 in males and females for titers of anti-KLH antibodies in a purebred White Leghorn layer line, corresponding estimates pooling all data for specific IgT, IgM, IgA and IgG were 0.12, 0.14, 0.10 and 0.07, respectively. Using the same genetic line, ref. [53] reported estimates of heritability for total antibody concentrations of IgT, IgM, IgA, and IgG of 0.08, 0.23, 0.22, and 0.06, respectively. Berghof et al. [33] documented the results from a study successfully increasing resistance to avian pathogenic *Escherichia coli* by selection for specific natural antibodies against KLH using the same WL line mentioned above [31]. Their results supported the hypothesis that levels of NAb might be used as an indicator trait to genetically improve disease resistance in egg-laying chickens. A more recent study described genetic and non-genetic factors affecting antibody response against KLH and generated in response to NDV vaccine on chicken populations from Africa [29]. Their heritability estimates for anti-KLH IgM, IgG, and IgA were 0.28, 0.14, and 0.07, respectively. Based on these results, they suggested that selection for genetic improvement of general and specific immunity was possible and would potentially improve disease resistance. However, the authors cautioned that an appropriate approach would require combining traits related to natural and acquired immunity in a multiple trait implementation as they found negative genetic correlations between KLH–nAbs and NDV–IgG of −0.26 to −0.9 [29]. 

Pathway analysis confirmed that genes with SNPs identified as associated with antibody levels were enriched with immune related protein classes. For joined analyses of NAbs and AAbs significant gene overrepresentations were found for RIR and WL breeds, with the top enriched classes being for defense/immunity protein and the immunoglobulin receptor superfamily. This result clearly indicates the alignment between the observed phenotypes, the genome-wide analyses, and the gene representations. Birds respond to stimulation with specific pathogens via vaccination against a variety of viral diseases (IBD, IVB, NDV and REO) or to other molecules known to interact with natural antibodies (KLH, OVA and PHA) by triggering genes responsible for coding the immune-related proteins. However, when gene enrichment was explored separately for NAbs and AAbs, similar gene overrepresentations were found for the RIR and WL breeds, mainly in terms of the expression of natural antibodies, which dominated the gene expression space. On the contrary, for WPR lines only significant overrepresentation of genes in response to induced antibodies were found. This result indicates that the nature of genetic responses against natural and induced antigens vary across lines of chickens selected for egg production.

## 5. Conclusions

Our results using eight lines from three breeds used in commercial egg production were consistent indicating that vaccine-induced and natural antibody responses were relatively higher in brown-egg lines (mainly WPR) than in white-egg lines (WL). Estimates of heritability for both types of response were highly variable, with large standard errors, but average estimates of heritability across lines were sizeable, which give an indication of the potential scope of use of these traits in breeding strategies by exploiting differences in immune responses between lines while, at the same time, putting selection pressure on the populations that show moderate heritability.

## Figures and Tables

**Table 1:** Descriptive statistics for antibody response as defined by SP ratios by line, sex, and generation.

FACTOR	n	IBD		IBV		NVD		REO		KLH		OVA		PHA	
Line		Mean	STDev	Mean	STDev	Mean	STDev	Mean	STDev	Mean	STDev	Mean	STDev	Mean	STDev
RIR1	720	2.77	1.136	1.43	0.805	5.55	1.29	1.64	0.684	0.88	0.643	1.53	1.083	0.61	0.347
RIR2	667	3.44	1.611	1.68	0.898	5.23	1.315	1.43	0.778	0.88	0.918	1.47	1.16	0.56	0.281
WL1	416	2.73	1.733	2.65	0.974	3.6	1.469	1.08	0.355	0.85	0.725	0.83	0.671	0.62	0.409
WL2	618	3.11	1.318	1.21	0.616	4.94	1.592	1.63	0.563	0.69	0.7	0.66	0.513	0.56	0.315
WL3	571	2.1	0.992	0.91	0.453	3.89	1.147	1.1	0.499	0.69	0.72	0.8	0.571	0.55	0.271
WL4	517	4.56	1.86	2.38	0.99	5.2	1.887	1.36	0.677	0.94	0.844	0.88	0.679	0.82	0.408
WPR1	319	3.19	1.29	3.13	1.114	5.55	1.013	1.62	0.613	1.14	0.982	1.92	1.354	0.64	0.283
WPR2	618	2.31	1.131	2.39	1.216	5.57	1.439	1.6	0.559	1.22	0.942	2.04	1.308	0.6	0.344
Sex															
Females	100	5.614	1.582	0.784	0.431	5.817	1.798	0.761	0.56						
Males	1597	2.96	1.514	1.852	1.103	4.882	1.614	1.507	0.642	0.91	0.833	1.264	1.09	0.621	0.345
Generation															
2017J	99	1.404	0.578	2.652	0.974	2.913	1.051	1.077	0.355	0.849	0.725	0.834	0.671	0.625	0.409
2018K	46	1.812	0.872	1.349	0.608	3.599	1.16	1.105	0.535	0.868	0.709	0.97	0.766	0.602	0.368
2018L	754	2.6	1.141	1.696	1.128	4.739	1.412	1.397	0.61	0.922	0.856	1.35	1.142	0.621	0.333
2019M	798	3.892	1.761	1.758	1.046	5.576	1.62	1.761	0.69						
		Number of samples		Number of samples		Number of samples		Number of samples		Number of samples		Number of samples		Number of samples	
NseqRIR ^1^		394		409		394		442		193		193		193	
NseqWL ^1^		769		568		723		477		381		381		381	
NseqRIR ^1^		368		287		288		288		185		185		185	

^1^ Nseq: number of samples with sequence and phenotypic information per breed.

**Table 2:** Estimates of variance components and heritability for anti-IBD antibody response.

			Pedigree				Genomic		
LINE	Animal	SE	Residual	SE	Heritability	SE	Heritability	SE	*p*-Value
RIR1	0.000	0.000	0.950	0.095	0.000	0.000	0.005	0.135	0.488
RIR2	0.080	0.216	1.427	0.246	0.053	0.141	0.068	0.118	0.254
RIRjoined	0.001	0.000	1.219	0.143	0.001	0.095	0.000	0.071	0.500
WL1	0.192	0.183	1.054	0.196	0.154	0.144	0.033	0.273	0.452
WL2	0.087	0.157	1.063	0.180	0.075	0.135	0.096	0.113	0.158
WL3	0.159	0.126	0.642	0.128	0.198	0.152	0.153	0.124	0.073
WL4	0.657	0.304	1.517	0.272	0.302	0.130	0.170	0.091	0.010
WL joined							0.000	0.047	0.500
WPR1	0.000	0.000	1.426	0.143	0.000	0.000	0.000	0.136	0.500
WPR2	0.113	0.185	1.085	0.199	0.094	0.153	0.207	0.140	0.052
WPRjoined							0.000	0.092	0.500

**Table 3:** Estimates of variance components and heritability for anti-IBV antibody response.

			Pedigree				Genomic		
LINE	Animal	SE	Residual	SE	Heritability	SE	Heritability	SE	*p*-Value
RIR1	0.06	0.050	0.33	0.052	0.15	0.125	0.08	0.099	0.199
RIR2	0.38	0.160	0.37	0.129	0.51	0.186	0.59 *	**0.147**	**0.000**
RIRjoined	0.17	0.062	0.36	0.057	0.32	0.111	0.29 *	**0.082**	**0.000**
WL1	0.00	0.000	0.95	0.135	0.00	0.000	0.00	0.274	0.500
WL2	0.07	0.063	0.30	0.063	0.19	0.169	0.00	0.107	0.500
WL3	0.12	0.042	0.09	0.032	0.58	0.167	0.46 *	**0.146**	**0.000**
WL4	0.00	0.000	0.98	0.139	0.00	0.000	0.00	0.245	0.500
WL joined							0.00	0.028	0.500
WPR1	0.00	0.000	1.24	0.176	0.00	0.000	0.00	0.313	0.500
WPR2	0.40	0.255	1.07	0.236	0.27	0.165	0.20 *	**0.135**	**0.041**
WPRjoined							0.00	0.107	0.500

* Heritability estimates in bold are significantly greater than 0.

**Table 4:** Estimates of variance components and heritability for anti-NDV antibody response.

			Pedigree				Genomic		
LINE	Animal	SE	Residual	SE	Heritability	SE	Heritability	SE	*p*-Value
RIR1	0.00	0.000	1.02	0.102	0.00	0.000	0.00	0.146	0.500
RIR2	0.25	0.200	1.00	0.202	0.20	0.154	0.13	0.128	0.123
RIRjoined	0.06	0.114	1.07	0.132	0.06	0.100	0.05	0.083	0.366
WL1	0.32	0.257	1.37	0.266	0.19	0.148	0.11	0.283	0.342
WL2	0.51	0.342	1.59	0.333	0.24	0.155	0.13	0.119	0.098
WL3	0.32	0.229	0.97	0.220	0.25	0.170	0.26 *	**0.142**	**0.026**
WL4	0.00	0.000	3.08	0.275	0.00	0.000	0.00	0.108	0.500
WL joined							0.00	0.033	0.500
WPR1	0.57	0.406	0.47	0.357	0.55	0.359	0.38 *	**0.274**	**0.054**
WPR2	0.01	0.245	1.83	0.298	0.01	0.135	0.00	0.116	0.493
WPRjoined							0.00	0.119	0.500

* Heritability estimates in bold are significantly greater than 0.

**Table 5:** Estimates of variance components and heritability for anti-REO antibody response.

			Pedigree				Genomic		
LINE	Animal	SE	Residual	SE	Heritability	SE	Heritability	SE	*p*-Value
RIR1	0.00	0.042	0.37	0.059	0.00	0.131	0.09	0.131	0.231
RIR2	0.12	0.068	0.33	0.064	0.27	0.141	0.22 *	**0.112**	**0.005**
RIRjoined	0.07	0.041	0.35	0.043	0.16	0.097	0.15 *	**0.078**	**0.012**
WL1	0.05	0.058	0.07	0.054	0.42	0.436	0.00	0.292	0.500
WL2	0.00	0.039	0.30	0.047	0.01	0.126	0.00	0.116	0.500
WL3	0.11	0.104	0.15	0.096	0.42	0.391	0.22	0.283	0.200
WL4	0.00	0.000	0.46	0.065	0.00	0.000	0.00	0.205	0.500
WL joined							0.00	0.031	0.500
WPR1	0.00	0.000	0.38	0.053	0.00	0.000	0.00	0.297	0.500
WPR2	0.00	0.000	0.30	0.030	0.00	0.000	0.00	0.132	0.500
WPRjoined							0.00	0.135	0.500

* Heritability estimates in bold are significantly greater than 0.

**Table 6:** Estimates of variance components and heritability for anti-KLH antibody level.

			Pedigree				Genomic		
LINE	Animal	SE	Residual	SE	Heritability	SE	Heritability	SE	*p*-Value
RIR1	0.00	0.000	0.41	0.058	0.00	0.000	0.00	0.323	0.500
RIR2	0.74	0.402	0.13	0.324	0.85	0.385	0.65 *	**0.262**	**0.005**
RIRjoined	0.14	0.177	0.48	0.171	0.22	0.281	0.25	0.182	0.116
WL1	0.00	0.000	0.53	0.075	0.00	0.000	0.01	0.246	0.484
WL2	0.00	0.000	0.49	0.070	0.00	0.000	0.00	0.240	0.500
WL3	0.00	0.000	0.52	0.074	0.00	0.000	0.00	0.247	0.500
WL4	0.00	0.000	0.71	0.100	0.00	0.000	0.00	0.253	0.500
WL joined							0.00	0.045	0.500
WPR1	0.20	0.283	0.77	0.284	0.21	0.288	0.14	0.255	0.258
WPR2	0.56	0.410	0.36	0.342	0.61	0.393	0.60 *	**0.302**	**0.039**
WPRjoined							0.14	0.183	0.500

* Heritability estimates in bold are significantly greater than 0.

**Table 7:** Estimates of variance components and heritability for OVA antibody response.

			Pedigree				Genomic		
LINE	Animal	SE	Residual	SE	Heritability	SE	Heritability	SE	*p*-Value
RIR1	0.42	0.334	0.63	0.308	0.40	0.300	0.23	0.234	0.114
RIR2	0.45	0.527	0.84	0.489	0.35	0.390	0.45 *	**0.289**	**0.050**
RIRjoined	0.39	0.283	0.76	0.270	0.34	0.237	0.23	0.178	0.116
WL1	0.00	0.000	0.45	0.064	0.00	0.000	0.00	0.300	0.500
WL2	0.00	0.113	0.26	0.083	0.00	0.290	0.24	0.272	0.156
WL3	0.00	0.000	0.33	0.046	0.00	0.000	0.00	0.281	0.500
WL4	0.01	0.155	0.45	0.170	0.02	0.352	0.31	0.234	0.066
WL joined							0.00	0.093	0.500
WPR1	0.00	0.000	1.83	0.260	0.00	0.000	0.00	0.271	0.500
WPR2	0.37	0.520	1.32	0.509	0.22	0.301	0.21	0.262	0.191
WPRjoined							0.00	0.181	0.500

* Heritability estimates in bold are significantly greater than 0.

**Table 8:** Estimates of variance components and heritability for PHA antibody response.

			Pedigree				Genomic		
LINE	Animal	SE	Residual	SE	Heritability	SE	Heritability	SE	*p*-Value
RIR1	0.03	0.043	0.09	0.041	0.25	0.343	0.46	0.287	0.071
RIR2	0.00	0.000	0.08	0.011	0.00	0.000	0.10	0.253	0.337
RIRjoined	0.00	0.023	0.10	0.024	0.03	0.228	0.10	0.173	0.364
WL1	0.00	0.000	0.17	0.024	0.00	0.000	0.00	0.298	0.500
WL2	0.01	0.035	0.09	0.035	0.13	0.348	0.22	0.265	0.178
WL3	0.00	0.000	0.07	0.010	0.00	0.000	0.00	0.247	0.500
WL4	0.00	0.000	0.17	0.023	0.00	0.000	0.00	0.219	0.500
WL joined							0.00	0.081	0.500
WPR1	0.00	0.000	0.08	0.011	0.00	0.000	0.00	0.321	0.500
WPR2	0.02	0.034	0.10	0.034	0.17	0.283	0.02	0.226	0.465
WPRjoined							0.00	0.197	0.500

**Table 9:** Enrichment analysis and genes for the Rhode Island Red (RIR) lines.

PHANTER Protein Class *Pooled Antibodies*	*Gallus gallus* Ref. No.	Number Enriched	Expected	Fold Enrichment	Sign (+/−)	Δ Raw *p*-Value	FDR
Defense/immunity protein	474	8	1.94	4.13	+	7.49 × 10^−4^	1.47 × 10^−1^
Immunoglobulin receptor superfamily	231	5	0.94	5.30	+	2.75 × 10^−3^	2.71 × 10^−1^
Antimicrobial response protein	26	2	0.11	18.82	+	5.77 × 10^−3^	3.79 × 10^−1^
*Natural Antibodies*							
Immunoglobulin receptor superfamily	222	49	17.56	2.79	+	3.18 × 10^−11^	6.26 × 10^−9^
Defense/immunity protein	468	63	37.01	1.70	+	2.76 × 10^−5^	1.81 × 10^−3^
G-protein coupled receptor	390	62	30.84	2.01	+	1.10 × 10^−7^	1.09 × 10^−5^
Transmembrane signal receptor	777	94	61.45	1.53	+	3.10 × 10^−5^	1.53 × 10^−3^
oxidoreductase	542	22	42.68	0.51	-	3.52 × 10^−4^	1.39 × 10^−2^
Chaperone	186	4	14.71	0.27	-	1.45 × 10^−3^	4.76 × 10^−2^

**Table 10:** Enrichment analysis and genes for the White Leghorn (WL) lines.

PHANTER Protein Class *Pooled Antibodies*	*Gallus gallus* Ref. No.	Number Enriched	Expected	Fold Enrichment	Sign (+/−)	Δ Raw *p*-Value	FDR
Immunoglobulin receptor superfamily	231	27	8.14	3.32	+	2.41 × 10^−7^	4.76 × 10^−5^
Defense/immunity protein	474	31	16.70	1.86	+	1.64 × 10^−3^	1.61 × 10^−1^
Protein modifying enzyme	1354	69	47.70	1.45	+	2.91 × 10^−3^	1.91 × 10^−1^
*Natural antibodies*							
Immunoglobulin receptor superfamily	222	14	1.83	7.66	+	4.34 × 10^−9^	8.55 × 10^−7^
Defense/immunity protein	468	16	3.85	4.15	+	1.61 × 10^−6^	1.58 × 10^−4^
C2H2 zinc finger transcription factor	256	10	2.11	4.75	+	5.23 × 10^−5^	2.57 × 10^−3^
Zinc finger transcription factor	347	12	2.86	4.20	+	3.07 × 10^−5^	2.02 × 10^−3^

**Table 11:** Enrichment analysis and genes for the White Plymouth Rock (WPR) lines.

PHANTER Protein Class	*Gallus gallus* Ref. No.	Number Enriched	Expected	Fold Enrichment	Sign (+/−)	Δ Raw *p*-Value	FDR
C2H2 zinc finger transcription factor	256	6	0.98	6.15	+	4.29 × 10^−4^	4.22 × 10^−2^
Zinc finger transcription factor	347	7	1.32	5.29	+	3.48 × 10^−4^	6.85 × 10^−2^

## Data Availability

Restrictions apply to the availability of these data. Data were obtained from Hy-Line Int. and are available from the authors with the permission of Hy-Line Int upon a reasonable request.

## Supplementary Materials

The following supporting information can be downloaded at https://www.mdpi.com/article/10.3390/ani14111623/s1. Figure S1. Antibody levels against Infectious Bronchitis Disease (IBD) for different generations (2017J thru 2019M) and line-sex combinations. Figure S2. Antibody levels against IBV for different generations (2017J thru 2019M) and line-sex combinations. Figure S3. Antibody levels against New Castle Disease Virus (NVD) for different generations (2017J thru 2019M) and line-sex combinations. Figure S4. Antibody levels against Reovirus (REO) for different generations (2017J thru 2019M) and line-sex combinations. Figure S5. Natural antibodies binding keyhole limpet hemocyanin (KLH) for males of different generations (2017J thru 2018L) and lines. Figure S6. Antibody levels against ovalbumin OVA for males of different generations (2017J thru 2018L) and lines. Figure S7. Antibody levels against phytohemagglutinin (PHA) for males of different generations(2017J thru 2018L) and lines. Figure S8. QQ plots for antibody levels against IBD of WPR (A), RIR (B) and WL (C) lines. Figure S9. QQ plots for antibody levels against IBV of WPR (A), RIR (B) and WL (C) lines. Figure S10. QQ plots for antibody levels against KLH of WPR (A), RIR (B) and WL (C) lines. Figure S11. QQ plots for antibody levels against NDV of WPR (A), RIR (B) and WL (C) lines. Figure S12. QQ plots for antibody levels against OVA of WPR (A), RIR (B) and WL (C) lines. Figure S13. QQ plots for antibody levels against PHA of WPR (A), RIR (B) and WL (C) lines. Figure S14. QQ plots for antibody levels against REO of WPR (A), RIR (B) and WL (C) lines.
