# Supplementary material for: Genetic Variation in Natural and Induced Antibody Responses in Layer Chickens
Source: Animals (Basel). 2024 May 30;14(11):1623. doi: 10.3390/ani14111623 (PMC11171384; doi:10.3390/ani14111623)
Supplement: Supplementary file 1 [file animals-14-01623-s001.zip › animals-3015580-supplementary.pdf]

animals

MDPI

Supplementary Materials:

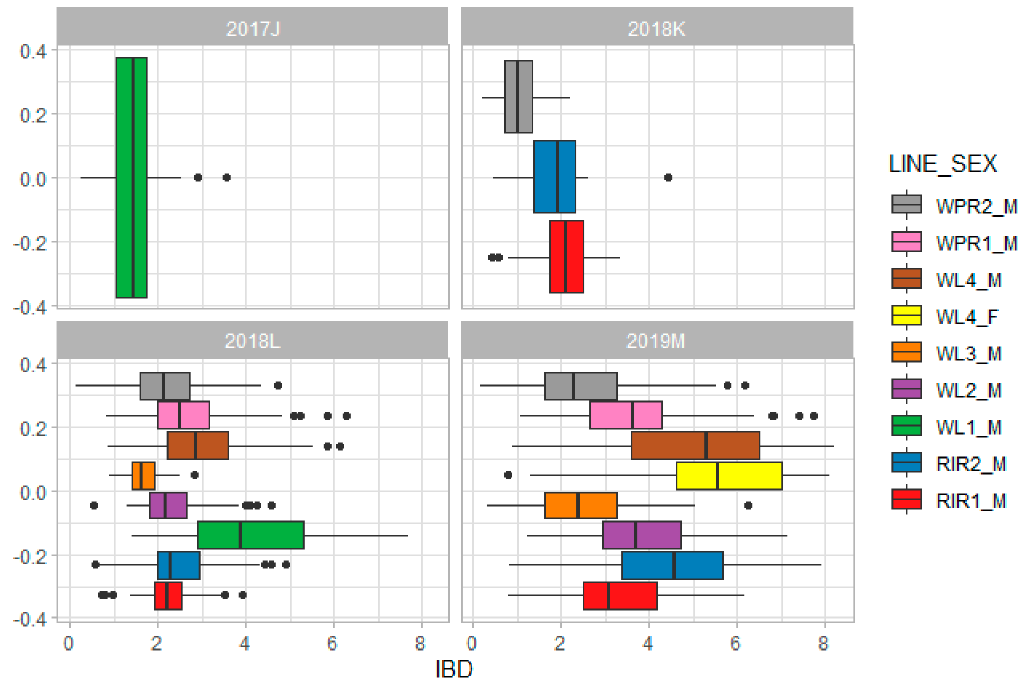

Figure S1. Antibody levels against Infectious Bronchitis Disease (IBD) for different generations (2017J thru 2019M) and line-sex combinations

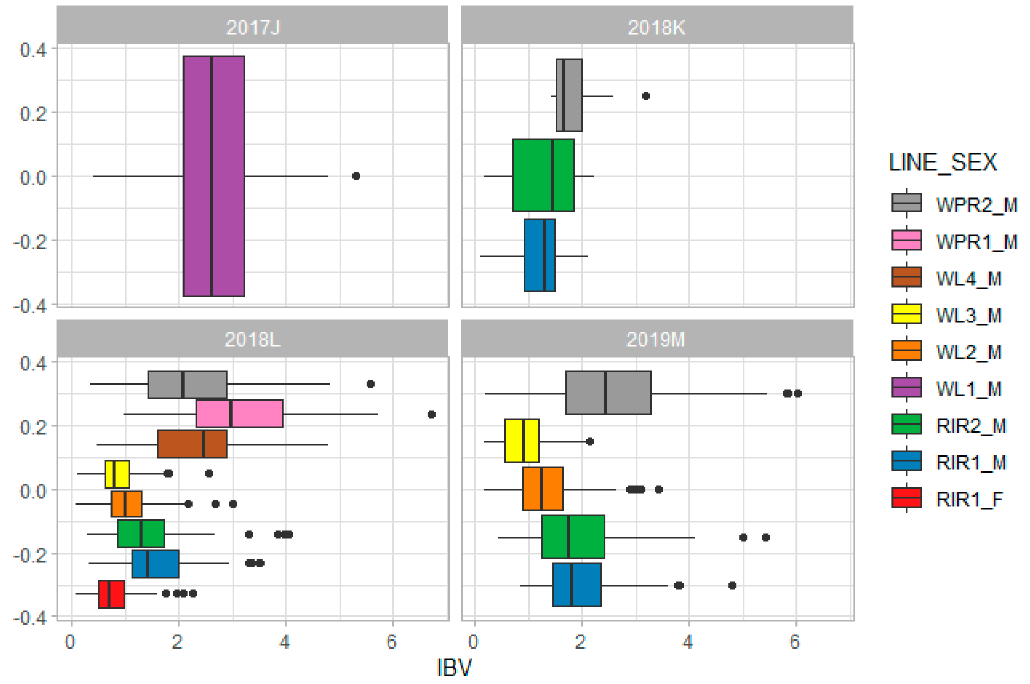

Figure S2. Antibody levels against IBV for different generations (2017J thru 2019M) and line-sex combinations

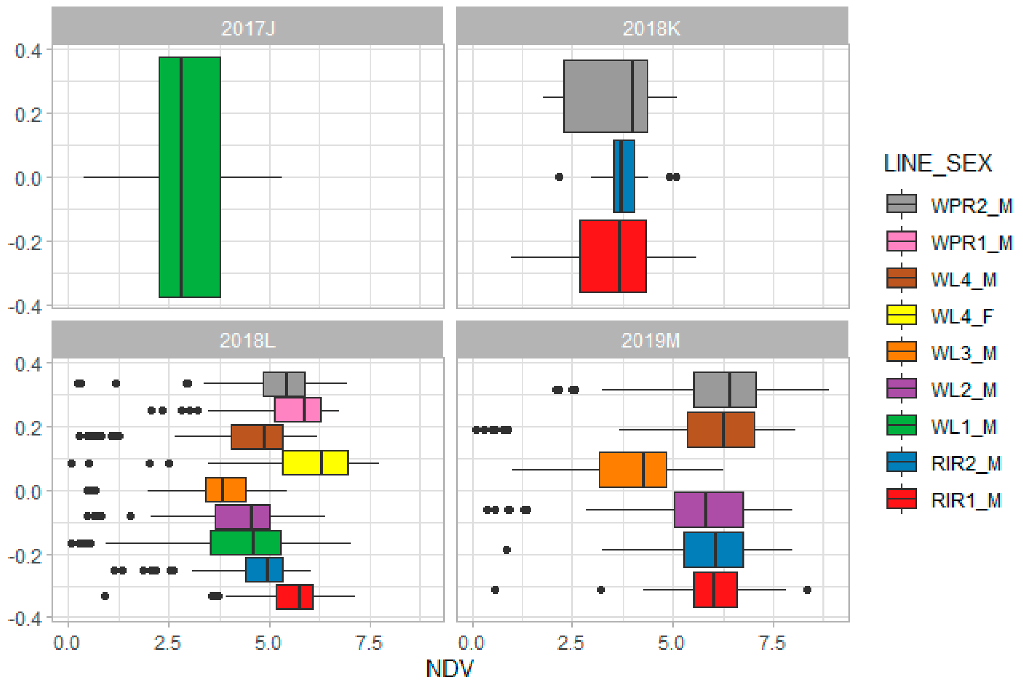

Figure S3. Antibody levels against New Castle Disease Virus (NVD) for different generations (2017J thru 2019M) and line-sex combinations

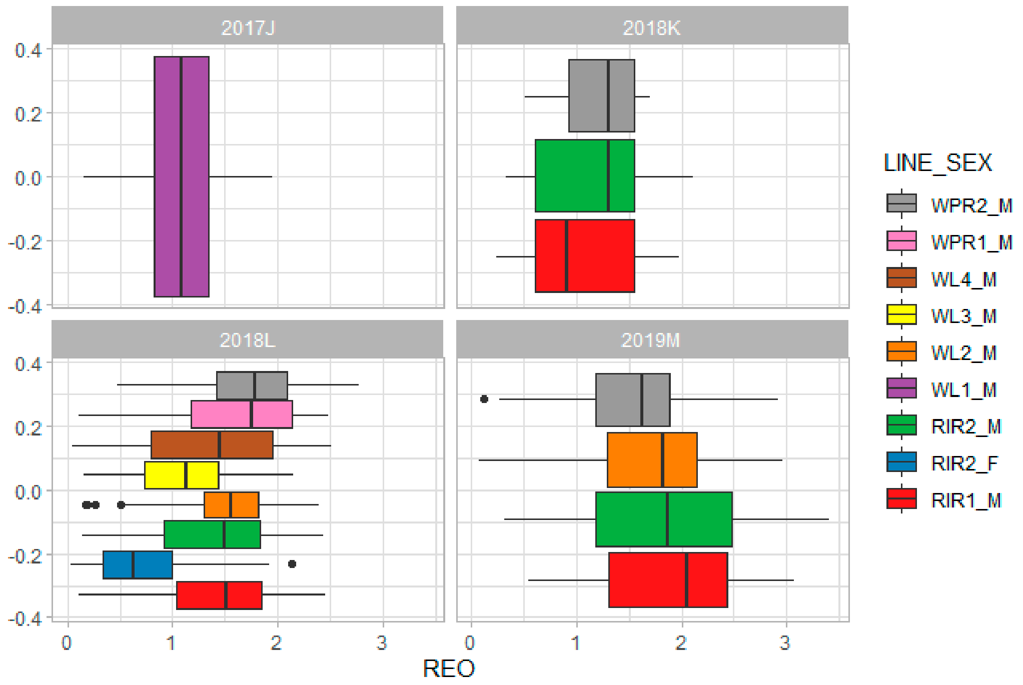

Figure S4. Antibody levels against Reovirus (REO) for different generations (2017J thru 2019M) and line-sex combinations

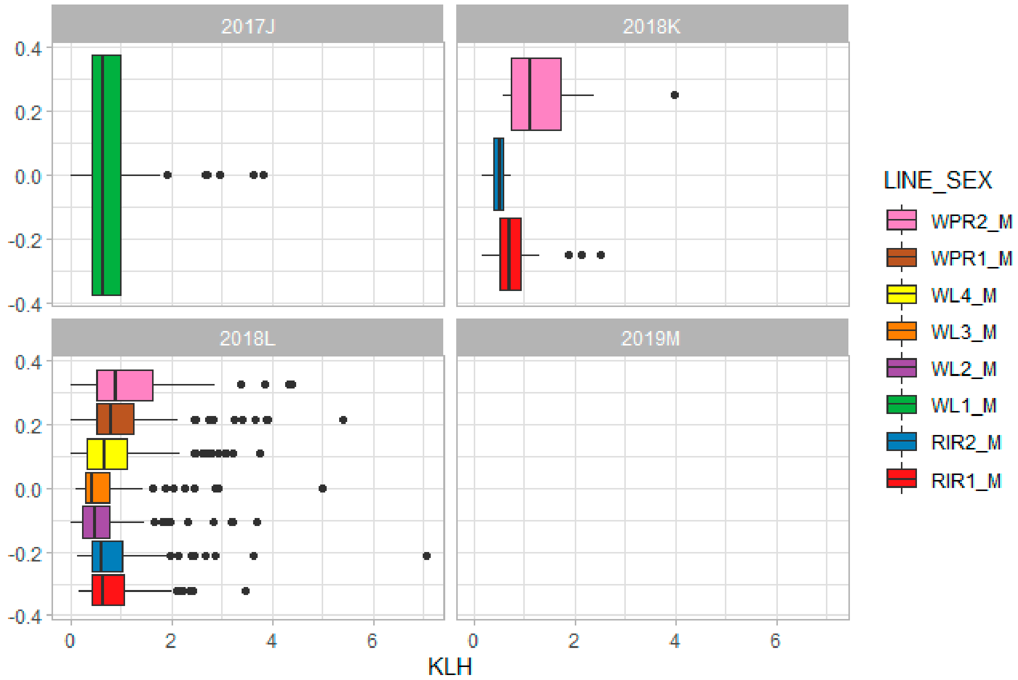

Figure S5. Natural antibodies binding keyhole limpet hemocyanin (KLH) for males of different generations (2017J thru 2018L) and lines

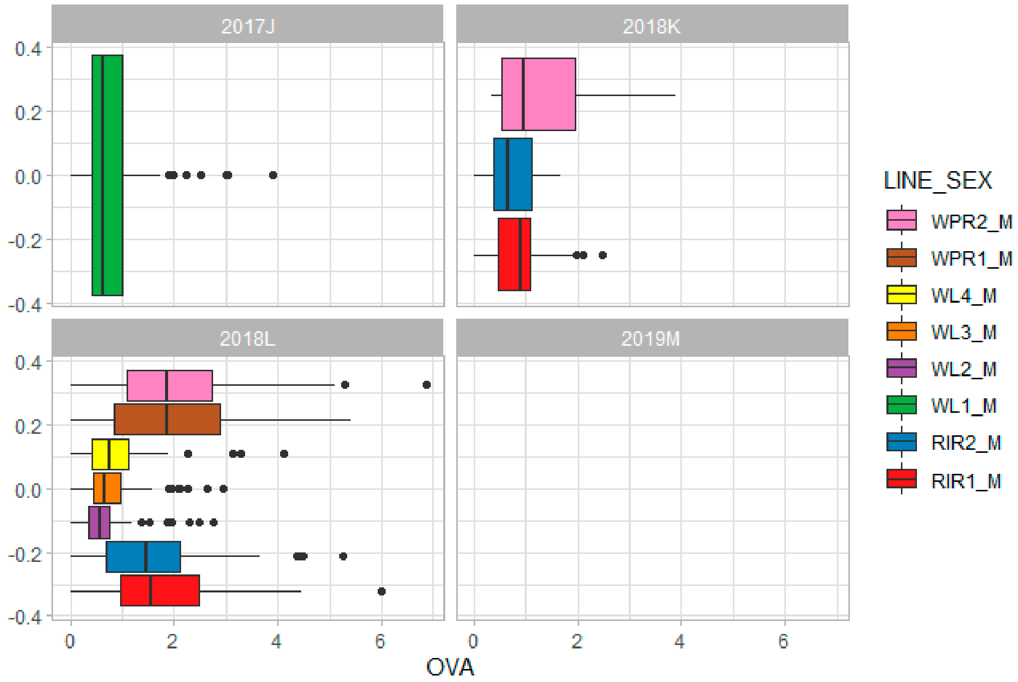

Figure S6. Antibody levels against ovalbumin OVA for males of different generations (2017J thru 2018L) and lines

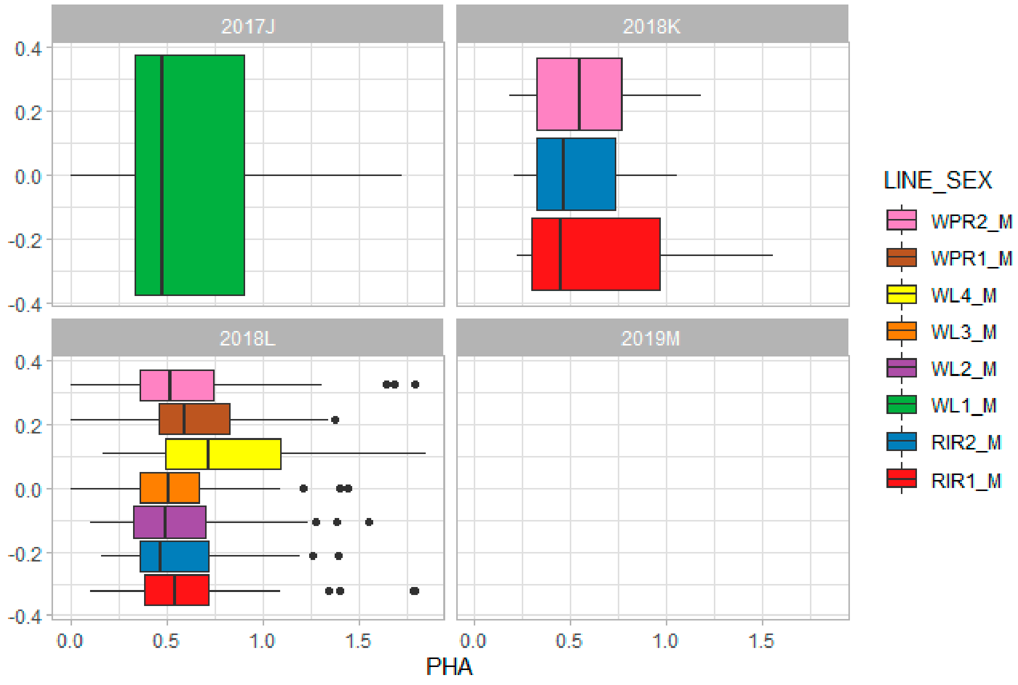

Figure S7. Antibody levels against phytohemagglutinin (PHA) for males of different generations(2017J thru 2018L) and lines.

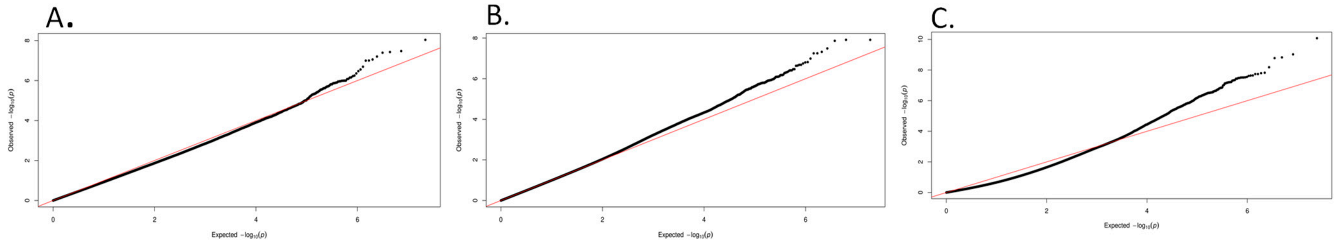

Figure S8. QQ plots for antibody levels against IBD of WPR (A), RIR (B) and WL (C) lines.

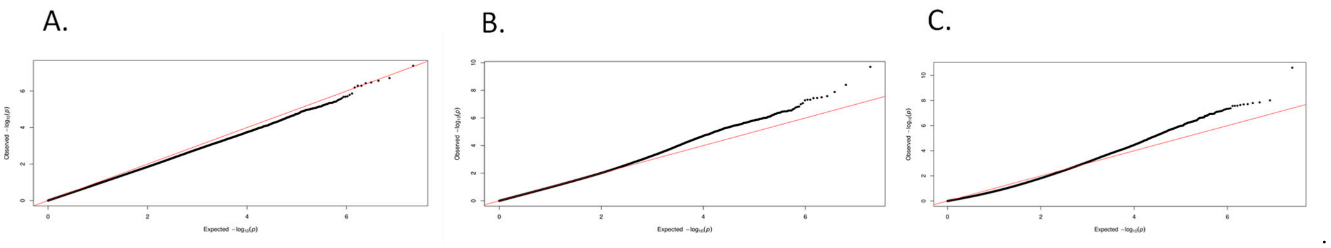

Figure S9. QQ plots for antibody levels against IBV of WPR (A), RIR (B) and WL (C) lines.

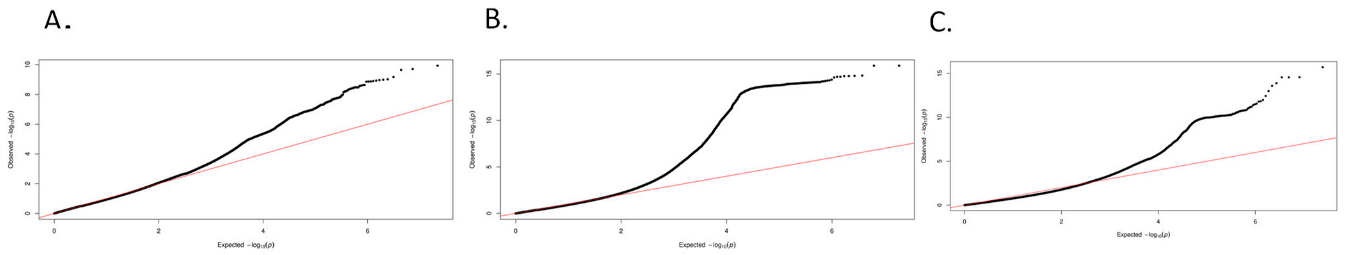

Figure S10. QQ plots for antibody levels against KLH of WPR (A), RIR (B) and WL (C) lines.

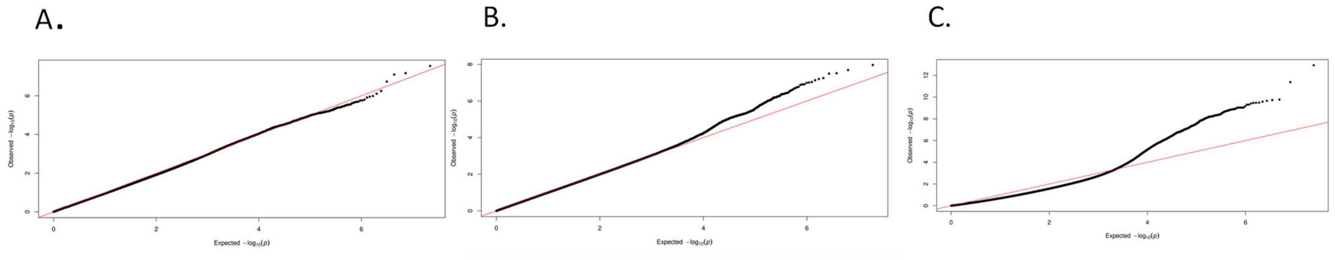

Figure S11. QQ plots for antibody levels against NDV of WPR (A), RIR (B) and WL (C) lines.

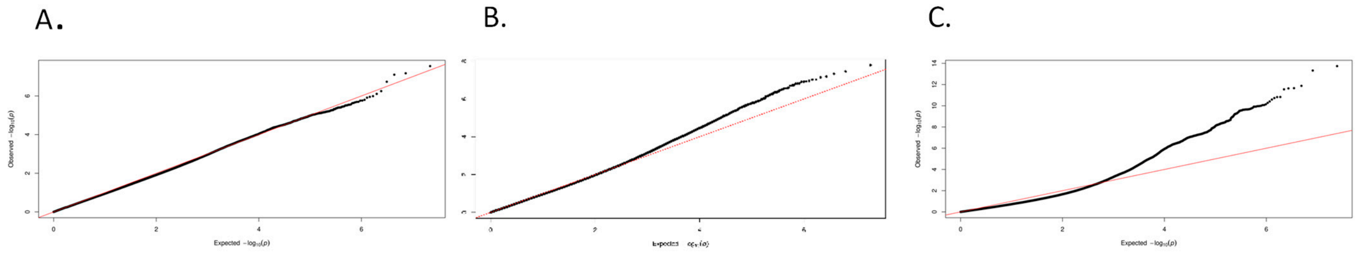

Figure S12. QQ plots for antibody levels against OVA of WPR (A), RIR (B) and WL (C) lines.

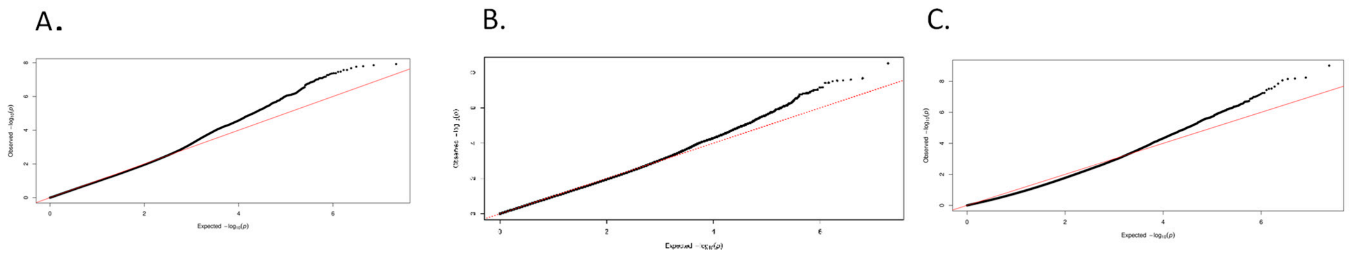

Figure S13. QQ plots for antibody levels against PHA of WPR (A), RIR (B) and WL (C) lines.

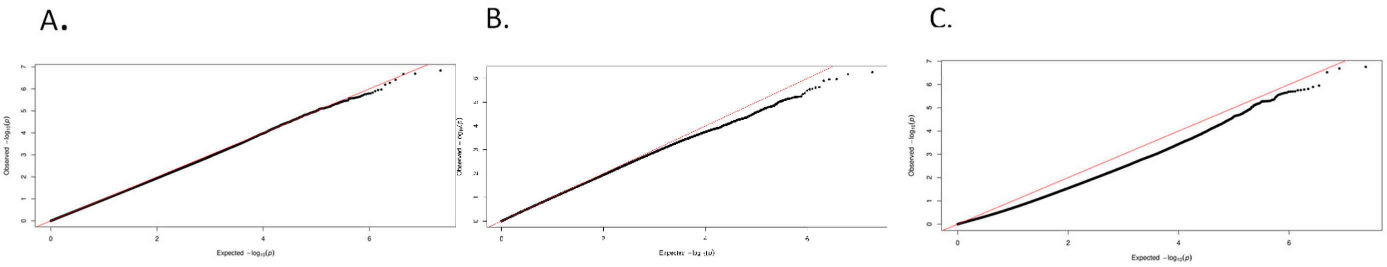

Figure S14. QQ plots for antibody levels against REO of WPR (A), RIR (B) and WL (C) lines.
